# Habitat Re-Creation (Ecological Restoration) as a Strategy for Conserving Insect Communities in Highly Fragmented Landscapes

**DOI:** 10.3390/insects4040761

**Published:** 2013-12-05

**Authors:** John A. Shuey

**Affiliations:** The Nature Conservancy of Indiana, 620 E Ohio Street, Indianapolis, IN 46202, USA; E-Mail: jshuey@tnc.org

**Keywords:** remnant-dependent insects, ecosystem restoration, insect conservation, climate change adaptation

## Abstract

Because of their vast diversity, measured by both numbers of species as well as life history traits, insects defy comprehensive conservation planning. Thus, almost all insect conservation efforts target individual species. However, serious insect conservation requires goals that are set at the faunal level and conservation success requires strategies that conserve intact communities. This task is complicated in agricultural landscapes by high levels of habitat fragmentation and isolation. In many regions, once widespread insect communities are now functionally trapped on islands of ecosystem remnants and subject to a variety of stressors associated with isolation, small population sizes and artificial population fragmentation. In fragmented landscapes ecological restoration can be an effective strategy for reducing localized insect extinction rates, but insects are seldom included in restoration design criteria. It is possible to incorporate a few simple conservation criteria into restoration designs that enhance impacts to entire insect communities. Restoration can be used as a strategy to address fragmentation threats to isolated insect communities if insect communities are incorporated at the onset of restoration planning. Fully incorporating insect communities into restoration designs may increase the cost of restoration two- to three-fold, but the benefits to biodiversity conservation and the ecological services provided by intact insect communities justify the cost.

## 1. Introduction

Insects, by their very nature, defy comprehensive conservation planning. Their vast diversity, measured by both numbers of species as well as life history traits, preclude detailed knowledge of the status and distribution of all but a few species. Because of this, almost all insect conservation efforts are focused at the species level. But serious insect conservation requires goals that are set at the faunal level and conservation success requires efforts that identify and conserve all species at all levels of biological organization. This seemingly daunting task is complicated in agricultural landscapes by high levels of habitat loss and fragmentation, resulting in population isolation. In many regions, once widespread insect communities are now functionally trapped on islands of suitable habitat and subject to a variety of stressors associated with isolation and small population sizes [1]. The threats from habitat fragmentation relative to conservation are well documented and entire books have been devoted to the subject (e.g., [2,3]). Fragmentation exacerbates a variety of threats to populations [4] and cumulatively, the ecosystems and the insect communities they support. 

In highly fragmented agricultural landscapes, regional insect communities are dominated by “landscape-dwelling” (or remnant independent) species that have adapted well to the anthropogenic landscapes that dominate most agricultural areas [1]. The members of these communities effectively utilize disturbed habitats composed of similarly tolerant plant species that dominate many agricultural and urban landscapes. The individual insect species composing the community are able to sustain populations across the human dominated landscape and for the most part, these individual species are not at risk for regional extirpation. 

However, hidden away within these broader fragmented landscapes there are hundreds, perhaps thousands of insect species that are incapable of surviving within the anthropomorphic matrix [1,5,6,7,8,9]. These species are closely tied to native ecosystem remnants that have escaped agricultural, industrial or urban landuse conversion. Typically persisting as small populations within small and widely dispersed remnants of once widespread habitat types, “remnant-dependent” (hereafter referred to as r-d) insect species comprise the bulk of the extinction-prone biodiversity surviving within this highly fragmented landscapes [1,10]. These insects characteristically have very narrow life history requirements that limit their potential distribution in disturbed habitats. For example, monophagous insects restricted to wetland adapted hostplants are unlikely find suitable conditions within disturbed, converted, or invasive species dominated habitats. Panzer *et al*. [9] estimate that in the wetlands, grasslands and savannas of northeast Illinois (USA), roughly 15% of all insect species may require ecosystems remnants and estimate that the total number of conservative insect species for this region exceeds 2,000 species. In highly fragmented landscapes, these r-d species comprise the bulk of insects expected to be threatened by regional population declines that could result in regional species extirpations. This community would benefit from conservation strategies that implicitly incorporate their needs.

Clearly, it is impossible to develop individual strategies that simultaneously address the threats to thousands of regionally imperiled insect species. Conservation strategies that use surrogate conservation targets such as representative terrestrial community types [11,12,13,14] can efficiently conserve the persisting habitats of many r-d insects [10,15], but do little to address the unique threats to insect communities imposed by extreme landscape fragmentation and resulting population isolation. Artificial population fragmentation and reduced habitat size increases the rate of population extinctions, producing impoverished insect communities, two of the factors that put r-d insect communities at risk in highly fragmented landscapes. While traditional small preserves may initially conserve a subset of the regional r-d community, over time individual species are likely to fade as isolated populations become extinct more rapidly than new populations are founded [16]. This problem is compounded in landscapes such is the Midwestern United States where over 99.9% of native grasslands have been converted to agriculture [17]. In Indiana, a typical prairie remnant may be 15 ha or less in extent, separated by 10 km or more from the nearest similar remnant, and supports small, vulnerable populations of many r-d insect species. 

While the real and theoretical impacts of habitat destruction relative to population dynamics in insects are well documented (e.g., [4,10,18,19]), very little research exists on the potential role habitat restoration and recreation could play in the conservation of r-d insect communities. When insect communities are considered within the context of habitat restoration, it is usually as an afterthought. Typically coarse ecological guilds or higher taxonomic groupings are compared between restorations and ecosystem remnants as a “measure of restoration success” [20,21,22]. These assessments are almost always focused at taxonomic levels that provide little insight into the population level dynamics that underpin the conservation of intact communities. Rather, these types of assessments assume that restoring the functional aspect of insect communities is indicative of “restoration success”, which certainly has validity if restoring ecosystem functions and processes were among the initial objectives of the restoration. But these coarse assessments shed no light on the potential benefits to r-d species and communities or how habitat restoration may reduce threats to r-d insect communities. 

Well designed and managed restorations can increase habitat for r-d insects [23,24,25]. But while criteria for size and structural components of restoration design criteria abound for vertebrates, especially amphibians and birds (e.g., [3,26,27]), r-d insects and communities are rarely incorporated into restoration design criteria. When insects are included in restoration planning it is limited to one or two endangered species [28,29,30,31,32]. Such species focused efforts, as important as they are, are more related to species-level recovery and are not considered further herein.

**Figure 1:**
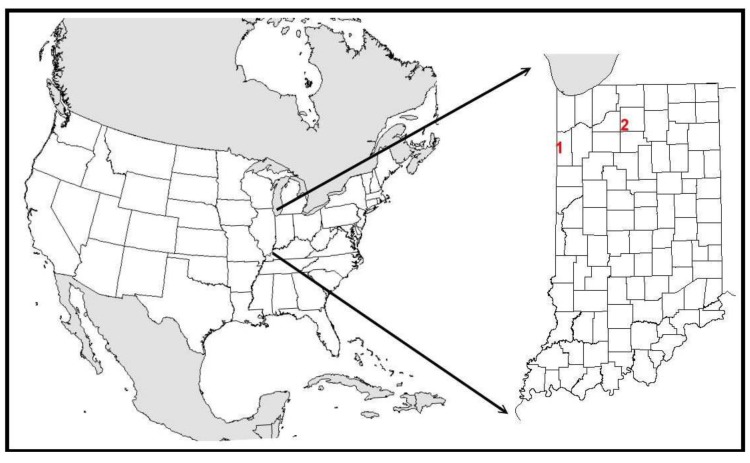
The locations of restorations at Kankakee Sands (1) and Houghton Lake (2). Both sites are located within the state of Indiana in North America.

The practice and theory of restoration ecology is well advanced, but biased towards restoring ecosystem functions and processes (e.g., [33,34]). Insect species are typically considered as members of ecological guilds, and once classified, individual species become “redundant”. Granted, increasing ecological redundancy is good [33,34,35], but by and large, there is no appreciation for the conservation of declining r-d insect communities within the overarching framework of restoration. Insect community response is relegated to the “if you build it, they will come” model. Of course, many species of insects will be present on all restorations, but the species that respond readily to this restoration philosophy are primarily members of the landscape dwelling community that thrives in the surround anthropomorphic matrix. The r-d community, whose species would most benefit from restoration, are typically ignored. Here, I address how habitat restoration and habitat recreation (hereafter simply referred to as restoration) can be used as a generalized strategy to stabilize regional declines in r-d insect communities in highly fragmented landscapes. Because conservation and restoration require site-based actions, I explain how these generalized criteria were incorporated into two very different restorations in Northern Indiana, USA (Figure 1).

## 2. Generalized Conservation Threats to Remnant-Dependent Insect Communities in Highly Fragmented Landscapes

Before any effective conservation strategy can be developed and implemented, it is imperative that it is placed within the context of threat reduction [36]. Threats to regional insect communities are always site specific and effective conservation actions address site-specific threats. However, r-d insects in highly fragmented landscapes are exposed to several general threat types that can be addressed by habitat restoration. While developing and implementing conservation strategies on a species by species is not practical, it is possible to conceptualize restoration strategies that have broad benefits to r-d species, both known and unknown, in fragmented landscapes. 

### 2.1. Small Population Size

Small populations are generally at a greater risk of extinction than large populations [37]. In highly fragmented landscapes, small patches of remnant habitat may support populations that are below thresholds that protect from loss of genetic variability and related problems of inbreeding and genetic drift, population fluctuations due to variations in birth and death rate, environmental fluctuations due to variation in predation, competition, disease and food supply, and natural catastrophes that occur at irregular intervals. 

Restoration can be used to increase habitat size and heterogeneity, increasing population size for individual species, reducing the threat from stochastic events. Restoration of complex habitat mosaics across environmental gradients can increase ecological resilience to environmental fluctuations and catastrophic events. In order to produce expanded habitat, restorations must include all (or most) requisite resources for remnant restricted insects, both known and unknown.

### 2.2. Population Isolation/Disrupted Population Dynamics

Fragmented landscapes force an artificial metapopulation structure on plants and animals that are restricted to ecosystem remnants. Despite most species having wings, many insects are not adept at navigating across converted landscapes (e.g., [5,38]) and for many species gene flow or recolonization of unoccupied habitat is unlikely. Metapopulation models predict that isolated populations are more likely to go extinct in the long run than populations that are slightly or well-connected [4]. In intact landscapes, declining populations can be “rescued” by immigration from a nearby expanding population. In fragmented landscapes, the distance between fragments may prevent this from happening resulting in low levels of habitat occupancy [4] and as a result, isolated ecosystem remnants generally support a subset of regional r-d insect communities. 

Restoration can be used to expand occupied habitat to the point that connectivity is enhanced or reestablished between ecosystem remnants. Stepping stone models or traditional corridors [29,39] can be used to enhance connectivity, but restoration of contiguous habitat may be required to enhance connectivity for insects with limited dispersal abilities [40]. Intuitively, restoration strategies that assume limited dispersal capabilities will accommodate more species than will stepping-stone models [39]. To influence connectivity, restorations must include requisite resources for remnant restricted insects throughout the intervening expanse if artificial metapopulations are to be restored to patchy or contiguous population structures.

### 2.3. Inappropriately Scaled Disturbance Regimes/Ecological Processes

The impacts to ecological communities from traditional ecological disturbances, such as fire, severe storms events and flooding are exacerbated by fragmentation, increasing the potential for local population declines or extinction. Even at sites managed to maintain ecological integrity, managed disturbance regimes such as prescribed fire can be implemented at scales that negatively impact populations [6,10,41]. Ironically, disturbance such as fire and seasonal flooding may be required to maintain suitable habitat for many r-d species, but these disturbances may negatively impact insect populations, increasing the probability of local population extinctions if there are no nearby source populations from which immigrants can recolonize recently disturbed habitats [10,41]. Similarly, ecological processes such as seasonal flooding or nutrient loadings may alter plant communities, resulting in declines of sensitive habitats [42] and the r-d insects they support.

Restoration designs can be scaled to specifically accommodate ecological disturbance regimes. Restorations that increase connectivity across landscapes that are impacted asynchronously by disturbances are likely to enhance recolonization following local extinction events. Increasing habitat size allows disturbances to be managed to create refugia from negative impacts, reducing the probability that disturbances produce local extinctions. Likewise, restorations can be designed to influence site hydrology and nutrient cycling. 

### 2.4. Future Predicted Climate Regimes

Future climates in many regions are expected to diverge from current climatic regimes [43], increasing negative stress to isolated insect populations. Small ecosystem remnants that persist in many highly fragmented landscapes typically have reduced environmental complexity as well, and do not effectively buffer habitats and species against changing climate. Predicted changes in temperature and the seasonality of precipitation will likely have dramatic impacts to isolated ecosystem remnants and the insects that they support in many regions. 

Restorations can be explicitly designed to improve local resilience to future predicted climatic regimes. For example, restorations can be used to restore connectivity between ecosystem remnants to provide access to nearby microhabitats and refugia that may buffer against climatic extremes. In many cases, restorations can be explicitly designed to increase local ecological heterogeneity in order to directly increase ecological resilience within and between restored habitats. It is also possible to design restorations to offset specific local threats from future predicted climatic regimes. 

## 3. Application of Generalized Restoration Strategies at Real-World Conservation Sites

It is critical to remember that the above strategies address a subset of conservation goals likely to be established for any given restoration. The threats to other conservation targets also influence restoration criteria to a greater or lesser extent. For example, vertebrate populations are likely to influence the scale of restoration more so than will r-d insect communities. I have not encountered a situation where r-d insect community criteria conflicted directly with other restoration goals. The following two examples highlight r-d insect community restoration at very different sites and scales. It is important to remember for both examples that the design criteria and goals presented here address a subset of the larger restoration goals at the sites. Both restorations incorporate strategies designed to reduce threats to imperiled vertebrate and vascular plant populations as well. 

### 3.1. Kankakee Sands

**Figure 2:**
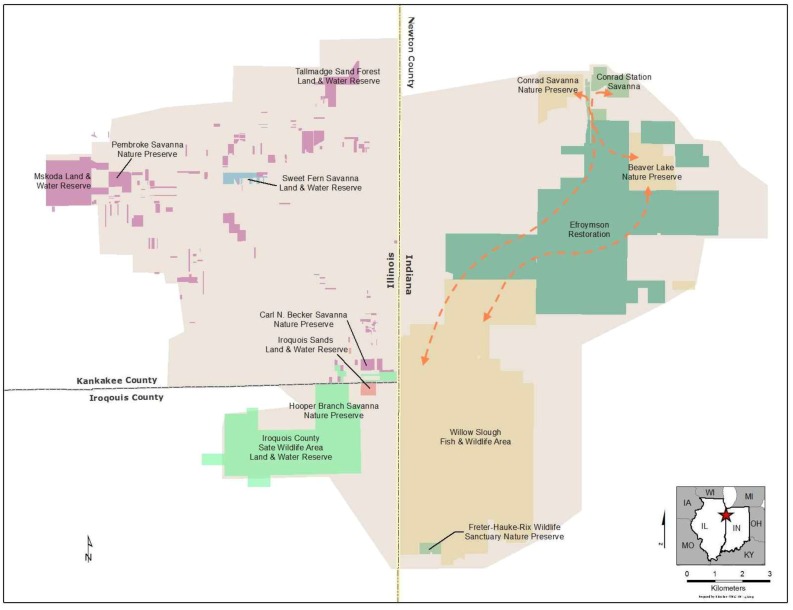
An overview of conservation lands at Kankakee Sands in Indiana and Illinois, USA. The Efroymson Restoration targeted agricultural lands that once restored, would restore connectivity between the primary Indiana Conservation areas.

The Efroymson Restoration at Kankakee Sands was conceived as a site-based strategy to alleviate the primary factors impacting a series of globally significant prairie and savanna remnants in northwestern Indiana [44]. As such, the restoration is designed to address broad viability concerns of entire communities that persist on the adjoining ecosystem remnants. The site has been extensively evaluated for r-d insects, and over 230 species are known from the project area [9]. The restoration goal was not to create a large sand-prairie complex, but rather to enhance the viability of the adjacent ecosystem remnants and all the native species found at in those remnants by reducing threats to community viability. The conservation area extends westward into Illinois as well (Figure 2) but this paper focuses exclusively on the restoration in Indiana. 

#### 3.1.1. Site Description

Three significant ecosystem remnants, Conrad Savanna, Beaver Lake Prairie, and Willow Slough State Fish and Wildlife Area (Figure 2) clustered in north Newton Co., Morocco, IN, USA are reasonably close to one another, but were ecologically isolated from each other by surrounding agricultural lands. Each of these remnants are managed for biodiversity and all are known to support rich assemblages on r-d insects [9,45]. However, an initial analysis of threats to long-term viability of the ecological communities at these sites indicated that isolation and reduced habitat size were the greatest long-term threats to these r-d insect communities and their habitats. 

Conrad Savanna is a 325 ha black oak sand savanna complex located 3km northwest of Beaver Lake Prairie, a 260 ha dry/mesic sand prairie remnant. Willow Slough State Fish and Wildlife Area is a 4,000 ha. mosaic managed for hunting, and contains a mix of black oak savanna, wet/mesic sand prairie, patches of pin oak flat-woods, as well as managed wetlands. 

**Figure 3:**
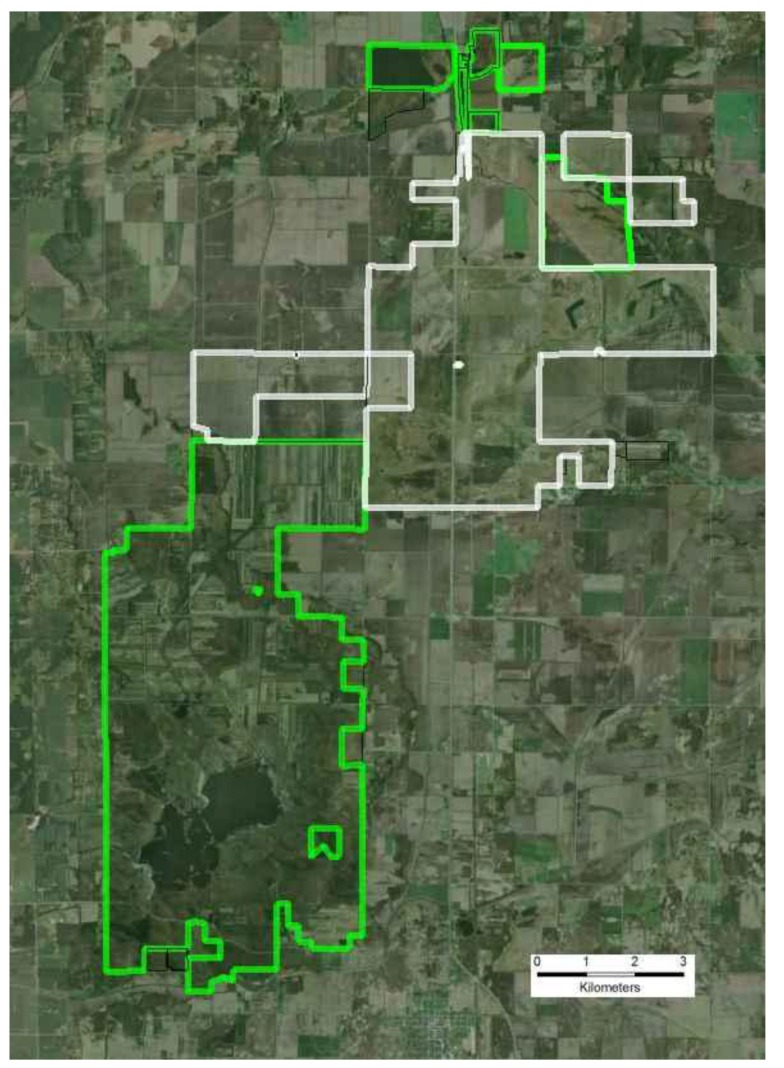
Air Photo of the Efroymson Restoration at Kankakee Sands demonstrating the dominance of agriculture in the region surrounding the restoration. The land conversion at the site is typical for many grain producing areas across the Midwestern United States. The restoration is outlined in white while pre-existing conservation areas are outlined in green.

Originally, these community types would have been part of a contiguous landscape mosaic, with black oak savanna on high sand dunes, grading down into dry, mesic, and wet prairie, with scattered pockets of deeper emergent wetlands. These community types share many plant species and several of the region’s vertebrates require more than one of these community types for their life cycle. Unfortunately, many critical community types persisted in near isolation on ecosystem remnants, surrounded by row-crop agriculture (Figure 3). A system of agricultural drainage ditches disrupted the pre-agricultural hydrology which was dominated by emergent wetlands and grasslands. Obvious and immediate threats to long-term viability of the individual remnants included: (1) small population size effects (reduced habitat size); (2) population isolation; and (3) altered ecological processes. In addition, future climatic regimes are expected to alter seasonality of temperature and precipitation patterns increasing the predicted frequency and duration of growing season droughts [43]. In 1996, The Nature Conservancy initiated a large-scale prairie restoration at the site, and each of these threats to r-d insects communities generated specific and explicit restoration goals for the project [44].

#### 3.1.2. Small Population Size

Many known r-d insects are limited to small habitat patches on the original ecosystem remnants ranging from wetlands to dry oak barrens [9,45]. We also assume that many additional but unknown insects are likewise habitat limited in the conservation area and that many of these species are monophagous. We developed four restoration design criteria to address these assumptions:

- Additional habitats adjacent to ecosystem remnants would be restored to increase available habitat for r-d insect communities. Note that the scale of this “restoration for habitat expansion for insects” was overridden by the demands for restoring connectivity at the site and by the habitat needs of density dependent grassland birds [26] and by the need to address connectivity across the site.
- Hydrology would be restored to capture the entire range of habitats used by r-d species. R-d insects at the site use habitats that range from xeric sand dunes to seasonally flooded wet prairie. Adjacent to the remnants, wetlands and mesic habitats had been drained to accommodate agricultural production. To the greatest extent possible we restored hydrology to bring back emergent wetland hydrology, allowing the restoration of a diverse range of habitats types across the entire hydrological gradient.
- The entire native plant community would be planted into the restoration. Because many r-d insect species are presumed to be monophagous, and we do not know hostplant requirements for all species, we decided to restore all native vascular plant species known from the three ecosystem remnants into the restoration. Seeds and plugs for 621 species have been planted in appropriate hydrologic zones to kick-start ecological healing for the entire botanical community in an attempt to establish hostplants for all potential r-d insects.
- Local genotype plant materials are exclusively used for the restoration. While this criterion is in part based on our desire to conserve local plant ecotypes, an underlying concern involves co-evolutionary relationships with native insect communities at regional scales. While we don’t know for certain if local insect populations are adapted to local plant populations, the conservative approach is to assure that we do not disrupt any local adaptations that may have evolved locally.

#### 3.1.3. Population Isolation/Disrupted Population Dynamics

The restoration is designed to create a contiguous complex mosaic of habitats that includes ecosystem remnants and restored habitats that function as a landscape of repeating habitats across ecological gradients [46]. By restoring the hydrologic gradient across the site and establishing a botanically diverse mosaic of prairie, savanna and wetland across the site, isolated populations should eventually expand to the point that artificial metapopulations are healed across the conservation area, thus altering the landscape dynamics into a more viable conservation area. 

#### 3.1.4. Inappropriately Scaled Disturbance Regimes

Interestingly, the early successional grassland habitats at Kankakee Sands are an anthropogenically maintained habitat. Grasslands first developed in the region during the Hypsothermal period, a period of higher temperatures and reduced precipitation that persisted between 6.00 and 4.00 BP [47,48]. The existing climate for the last two thousand years favors the development of forest habitats, but native Americans, using fire to manage habitats, maintained a high-diversity barrens/grassland/wetland mosaic that supports a high-diversity of native species in the region. Today, prescribed fire is still essential to manage succession in these habitats, but on small remnants, can result in population declines for some r-d insects. *The restoration is scaled to provide adequate unburned early successional habitat to accommodate planned management as well as to accommodate unplanned wildfires*. It is anticipated that over 1,800ha of restoration land will remain unburned annually, and that no more than 1/3^rd^ of habitat in existing ecosystem remnants will be burned annually. As the restoration heals, it is presumed that a more natural population structure that facilitates recolonization following local population extinctions will develop across the site for r-d insect species.

#### 3.1.5. Future Predicted Climate Regimes

**Figure 4:**
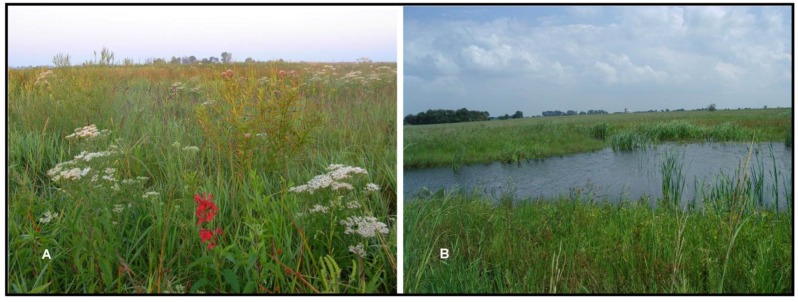
Two views of the restoration at Kankakee Sands demonstrating botanical diversity (**A**) and hydrologic complexity at the local scale (**B**). The restoration is explicitly designed to maximize ecological complexity as a strategy to increase potential habitat for r-d insects at the site, and to increase ecological resilience to potential climate change.

Within a 50 year horizon, the average annual temperature is predicted to increase by approximately 2 °C in Northern Indiana [43]. Annual precipitation is predicted to increase, but most of that increase will come during the dormant seasons. Late summer precipitation is expected to decrease, which when combined with higher predicted summer temperatures, will increase drought stress [43]. Although climate change will impact regional ecosystems in a variety of ways, it is likely that the most important direct impact of climate change will be drought stress [49], especially the occasional, but long-lasting and severe drought events that are more likely to occur under future predicted climate regimes [50]. Interestingly, conditions will resemble the Hypsothermal climatic conditions under which the grassland habitats of the region initially developed and thrived [49]. However, wetland and mesic habitats will likely be impacted during prolong droughts that lower the near-surface water table. To accommodate prolonged and severe drought, *hydrology will be restored to the maximum extent possible across the restoration to create wetland refugia across the site*. Where possible we have constructed a series of larger, open water wetlands that are more likely to maintain wetland habitats under predicted drought conditions. These wetland pockets will allow terrestrial habitats to adjust dynamically across the hydrologic gradient in response to cyclic climatic regimes. These open water wetlands range in size from 0.1 ha to 40 ha (Figure 4). In addition, these open water wetlands are expected to maintain important habitat for aquatic insect communities that are assumed to be primarily remnant-independent in the region. 

The Efroymson Restoration at Kankakee Sands is inherently different from the majority of prairie restoration in North America in its rationale and scale. The resulting restoration has created a block of contiguous habitat that now approaches 8,000 ha, including 3,200 ha of restored grassland and barrens designed explicitly to restore connectivity between ecosystem remnants for all extant species at the site. The first restoration units were planted in 1997 and planting will continue through at least 2016. Approximately 300 ha of additional agricultural land remains to be purchased in order to fully address criteria set forth in the restoration plan. It is worth noting that while the restoration is not solely designed to conserve r-d insects, incorporating the needs of r-d insects into the restoration criteria had a disproportionate impact on restoration design and cost. 

Although parts of the restoration are approaching 17 years post planting, a full assessment of the restoration has not yet been attempted. Preliminary results indicate that the restoration surrounding Beaver Lake Prairie supported 95 of the 140 r-d species known from that ecosystem remnant [51]. Similarly, an evaluation of leafhopper (Homoptera) diversity in the restoration found that higher plant richness led to 3- to 7-fold increases in leafhopper and r-d leafhopper species richness in restorations. Leafhopper community composition was more similar to remnant prairies in high richness than in low plant richness restorations [23]. These results provide intriguing insights into restoration performance at Kankakee Sands. The restoration is clearly serving as expansion habitat for some r-d insect species, in some cases far from removed from source populations, implying that connectivity is improving. A more detailed assessment of restoration performance is planned for 2014 and will focus on plant community establishment and landscape patterning, and restoration use by selected vertebrates (birds, amphibians and small mammals) and r-d insect communities (Lepidoptera, Hymenoptera, Coleoptera and Homoptera) relative to adjacent ecosystem remnants, degraded habitats and low diversity restoration plantings. 

### 3.2. Houghton Lake Wetlands

The restorations at Houghton Lake were conceived as a site-based strategy to alleviate the primary factors impacting a rare fen and natural lake complex located in Marshall County, Indiana, USA. The restorations were designed to address broad viability concerns of the entire community that persist at the site. Unlike Kankakee Sands, very little is known about potential r-d insects at the site but casual observations indicate that several species of r-d butterflies and moths occur at the site. In this region, fens are well known for the rich insect communities that they support, including many r-d species [9,52,53]. We presume that this fen is typical and our conservation goal is to enhance the viability of the wetland complex including all the native species these habitats support. 

#### 3.2.1. Site Description

**Figure 5:**
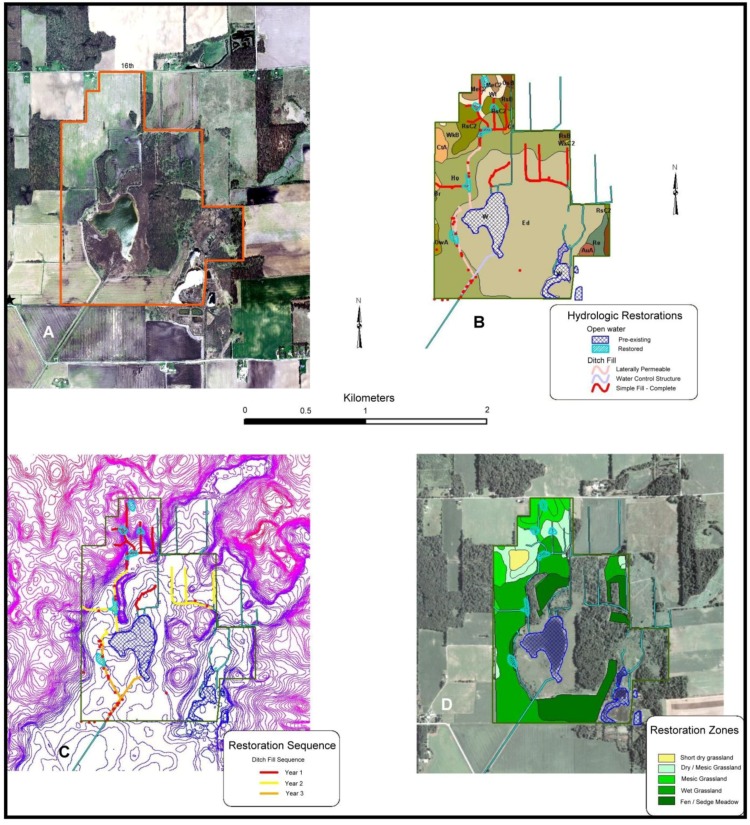
Houghton Lake restorations. The conservation site is embedded within a highly agricultural landscape (**A**) that required the installation of an extensive drainage network (**B**) of both open ditches and buried tiles to dry much soil in the wetland basin. These tiles diverted surface and subsurface water away from fen, sedge meadow and glacial lake habitats at the site. A detailed hydrologic restoration plan was developed (**C**) that enhanced groundwater flow to wetland habitats, which allow for the restoration of fen and sedge meadow habitats in wet muck soils in the wetland basin (**D**).

**Figure 6:**
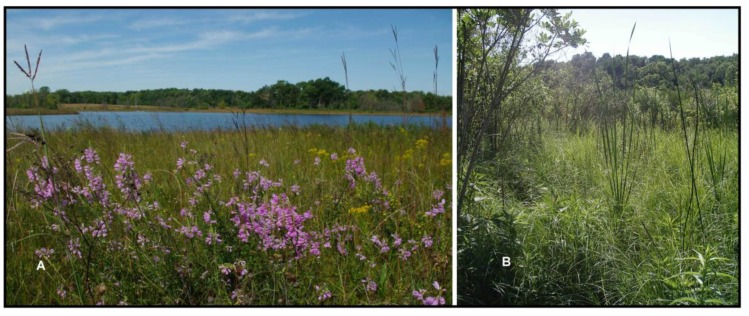
Houghton Lake Wetlands. Botanically rich marl flat wetlands surround much of the glacial lake (**A**) while sedge meadow (**B**) occurs over a groundwater upwelling to the east of the lake. These community types are presumed to support numerous r-d insect species at the site and are the focus of restoration efforts.

Houghton Lake is a small, isolated conservation area (Figure 5) composed of several naturally rare ecosystem remnants. Significant habitats include a small glacial lake (7.6 ha), adjacent marl-flat wetlands (16.4 ha) and a remnant fen (7.8 ha) (Figure 6). Fens and marl-flats are alkaline groundwater fed wetlands that support rich herbaceous communities. The lake and wetland complex drained southward through an extensive muck soil wetland complex that once supported extensive fen and sedge meadow habitat. The wetland basin was embedded within a woodland/forest/savanna mosaic over a rolling, glacial-till landscape. In this region, fens occur as discrete, small-patch communities dependent upon unusual hydrologic conditions [54], and the nearest similar wetlands were likely located 2–3 km distant along the edges of nearby glacial lakes. 

Today the basin is surrounded by agricultural land, and extensive drainage infrastructure has been installed to allow partial conversion of wetlands to row crop. A ditch was created through fen habitat to partially lower Houghton Lake and drain adjacent wetlands at the site for agricultural use. Some drained fen was eventually mined for marl, which was used to increase the alkalinity of the surrounding agricultural fields. These mined areas now support alkaline ponds surrounded by emergent wetlands. The nearest extant fen habitat remaining near Houghton Lake is approximately 3.5 km to the south east. 

Obvious threats to r-d insects at the Houghton Lake wetlands include: (1) small population size effects; (2) altered hydrology; and (3) inappropriately scaled ecological disturbances and processes. Note that because fens occur naturally as discrete, small patch habitats, habitat isolation is not viewed as a direct threat to viability for the wetland communities at the site. Nor, is it possible to create the appropriate geological settings that support fen habitat. Restoring connectivity to nearby fen habitat was not considered a threat reduction strategy that could be feasibly implemented.

From 2009 to 2010, we completed the initial phase of restoration to reduce threats to wetland communities at the site, including r-d insect communities. 

#### 3.2.2. Small Population Size

**Figure 7:**
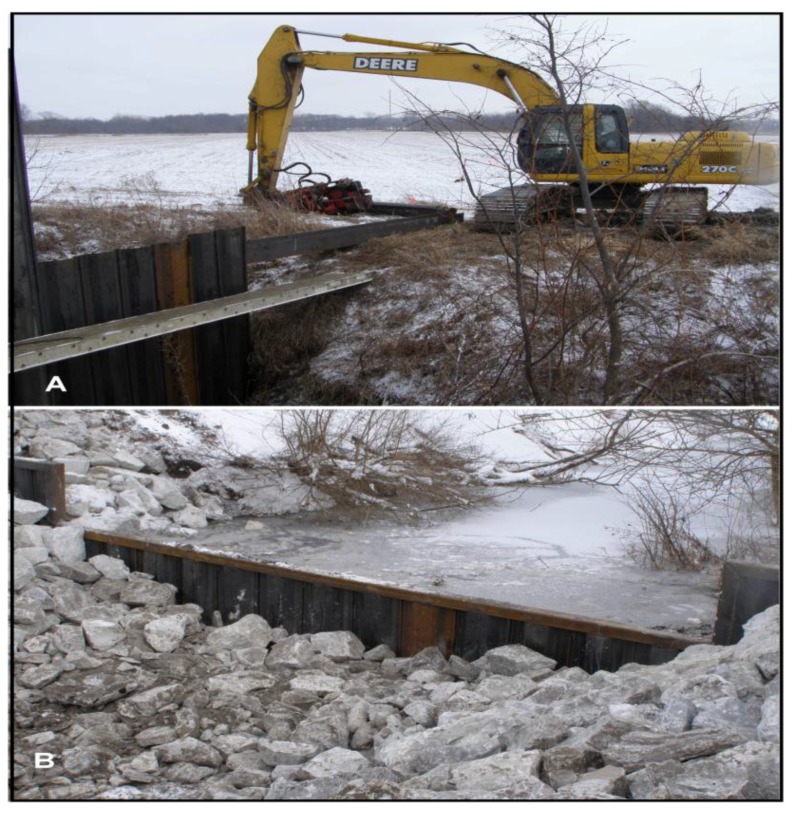
Houghton Lake Hydrologic restoration. As part of the hydrologic restoration, heavy equipment (**A**) was used to install a water control structure (**B**) across the man-made outflow to Houghton Lake. This allowed us to re-wet soils surrounding the southern portion of the lake to create suitable conditions for sedge meadow re-establishment.

The wetlands surrounding the lake have been reduced in extent by drainage and conversion to agriculture. We assume that the fens and emergent wetlands at the sites support r-d insects, and that habitat size has decreased population size for many some of these species. We developed four restoration design criteria to address these assumptions:

- *Hydrology would be restored to maximize groundwater flow into the lake basin and adjacent drained wetlands to restore wetland hydrology to adjacent drained muck soils*. Fens are fed by alkaline groundwater discharges, but agricultural practices intercepted groundwater and diverted it away from the lake and surrounding fields (Figure 5). As best as possible, groundwater flow was restored to enhance subsurface recharge of surrounding muck soils at the site in an attempt to recreate appropriate conditions for fen and sedge meadow restoration. In agricultural fields surrounding the wetland, we filled ditches and removed tile drainage to the maximum extent possible. This included creating upland basins which captured overland flow in order to increase infiltration of surface water flow into near-surface ground water flow. In addition, the Houghton Lake outfall was raised to help re-wet muck soils surrounding the wetlands (Figure 7).
- *For fen habitat, the entire native plant community would be seeded into the restoration*. Because the fen is assumed to support r-d species that are monophagous, we seeded as many of the known vascular plant species as possible into the restoration. This included over 110 species planted as seed over bare soil. Because sedges often do not establish well from seed, we planted approximately 10,000 plugs of late-successional rhizominous *Carex* species to aid in the initial establishment of sedge meadows habitat. The agricultural uplands surrounding wetlands were not restored as r-d insect habitat. Because no remnant upland grasslands persisted at the site, there was no concern for r-d insects in this habitat type. Uplands were restored to meet habitat criteria for regionally rare vertebrates known to occur at the site and to facilitate groundwater recharge. Uplands were planted at a relatively low cost to moderate diversity grasslands.
- *Local genotype plant materials are exclusively used for the restoration of fen habitats*. Similar to Kankakee Sands, we desire to conserve local plant ecotypes and potential coevolutionary relationships with native insect communities. Seeds for many species were collected on site from sedge meadow and fen for use in the adjacent restorations.

Our approach to restoring groundwater flow into the wetlands partially addressed ecological processes, but excess nutrients from adjacent agricultural fields was also a concern [42]. *Uplands that drain directly into Houghton Lake will be managed to reduce nutrient inputs from agriculture*. Nitrogen inputs disrupt nutrient poor fen communities, increasing the threat from invasive species. To reduce nutrient inputs to the lake, we restored much of the surface watershed that drains into the lake to grassland habitat. This eliminated agricultural nutrient inputs to fields located adjacent to the wetland basin. 

Fens are also fire adapted ecological systems [53], but in small ecological remnants, fire can result in population declines for some r-d insects. *The restoration is designed to maximize adequate unburned wetland habitat to accommodate planned fire management as well as to accommodate unplanned wildfires*. By restoring muck fields to the north and south of the lake to fen and sedge meadow, we have decreased the probability that more than 50% of r-d insect supporting habitat will be consumed by fire in any given growing season. This should allow for enhanced population recovery or recolonization of habitat following prescribed burning once the restoration is fully healed.

#### 3.2.3. Future Predicted Climate Regimes

The predicted future climate at Houghton Lake is similar to that for Kankakee Sands. Temperatures will increase and precipitation, while increasing on an annual basis, will decrease during the growing season increasing drought potential. Because the primary terrestrial habitats of conservation concern at the site are groundwater fed, we developed a strategy that will restore groundwater flow to the fen and lake. Once restored, we presume that predicted increases in autumn, winter and spring precipitation will adequately recharge regional near-surface aquifers, helping to ensure year-round groundwater flow to wetland habitats. Fens and glacial lakes in northern Indiana are known to have persisted during the Hypsothermal period [55] which is a natural climatic counterpart to predicted future climatic regimes and we presume that if groundwater flow is maintained, fen and sedge meadow habitat will persist at the site. 

Once fully restored, Houghton Lake Preserve will be a unique natural resource in Indiana–a fully protected small glacial lake, surrounded by a mosaic of natural and restored wetlands and grasslands. The resulting restorations have created a compact conservation area of 130 ha and an additional 15 ha remain to be purchased to complete the restoration design. Restorations include 14 ha of restored sedge meadow and fen wetland at the site, and another 31 ha of grasslands buffer much of the surface watershed that drains into the lake. At this time, the restoration plantings at Houghton Lake are in the initial stages of establishment (three to four years post planting) and succession remains very dynamic as desirable species compete with annual weeds and non-native species. The rich soils are vulnerable to invasive species invasion, and intensive invasive species management will be required for several more years to control potentially explosive problems. The wetland plantings are species rich, and rhizominous sedges are well established throughout appropriate habitats. At this point it is impossible to assess the restoration relative to r-d insects but we expect that by 2018, restored habitats will have stabilized to the point that an assessment for r-d species would be enlightening.

## 4. Conclusions

Everything we know about island biogeography and metapopulation dynamics points to the decline of r-d insect communities in highly fragmented landscapes. While some might argue that we can maintain at-risk species using intensive interventions such as facilitated dispersal [56] or population augmentations, at best these actions can target just a few, well known or charismatic species. Without effective strategies, the vast majority of r-d insects will continue to decline into obscurity.

The two examples highlighted here demonstrate the applicability of restoration as a conservation tool for conserving r-d species in highly fragmented landscapes. While it is possible to envision generalized threats to r-d insect communities in fragmented landscapes, threat reduction strategies must be adjusted to address site specific conditions. Implementing generalized strategies without incorporating site-specific realities will create inefficiencies that waste resources. For example, planting high-diversity grasslands in the uplands surrounding Houghton Lake would have doubled initial restoration costs of those agricultural fields, and there is no reason to believe that r-d species will particularly benefit from a more diverse upland restoration. Likewise, restoring connectivity to nearby habitats, the “universal conservation solution” in fragmented systems, may not be feasible or even required to significantly reduce threats in many situations. Regardless, habitat restoration can be an effective strategy at isolated sites reducing other stressors caused by habitat loss/fragmentation, especially threats from small population size and inappropriately scaled ecological disturbances. 

It is worth noting that ecological restoration is an expensive endeavor. Incorporating the needs of r-d insect communities significantly increases restoration cost. Restoring entire plant communities to increase habitat for known and unknown r-d insects may double or triple the cost for initial planting relative to traditional restorations which typically restore just 10%–20% of vascular plant diversity. Because local genotypes for the vast majority of species are not available commercially, the seed for the majority of plant species must be wild collected or grown in on-site nurseries. 

Are r-d insect communities worth the added cost and complexity required to incorporate into appropriate restoration designs in highly fragmented landscapes? Insects comprise the vast majority of regional biodiversity, and in fragmented landscapes, imperiled insects likely outnumber all other imperiled plants and animals combined. Insect communities provide ecosystem services including nutrient cycling, pest control, pollination, and wildlife nutrition [57]. It is time for entomologists to engage with restoration ecologists to ensure that our interests are met by the restorations of the future. Too often insect conservationists settle for the inexpensive solutions to threats, remaining too timid to seek real threat reduction strategies. Insects provide valuable ecological services [57] that are worthy of the conservation investment required to maintain intact insect communities. Alternatively, we can simply accept the increasing impoverishment of insect communities in fragmented landscapes, and maintain our status quo as the observers of ecological decline.
